# Functional connectivity in a rhythmic inhibitory circuit using Granger causality

**DOI:** 10.1186/2042-1001-1-9

**Published:** 2011-05-25

**Authors:** Tilman Kispersky, Gabrielle J Gutierrez, Eve Marder

**Affiliations:** 1 415 South Street, Biology Department and Volen Center, MS 013, Brandeis University, Waltham, MA 02454-9110, USA

## Abstract

**Background:**

Understanding circuit function would be greatly facilitated by methods that allow the simultaneous estimation of the functional strengths of all of the synapses in the network during ongoing network activity. Towards that end, we used Granger causality analysis on electrical recordings from the pyloric network of the crab *Cancer borealis*, a small rhythmic circuit with known connectivity, and known neuronal intrinsic properties.

**Results:**

Granger causality analysis reported a causal relationship where there is no anatomical correlate because of the strong oscillatory behavior of the pyloric circuit. Additionally, we failed to find a direct relationship between synaptic strength and Granger causality in a set of pyloric circuit models.

**Conclusions:**

We conclude that the lack of a relationship between synaptic strength and functional connectivity occurs because Granger causality essentially collapses the direct contribution of the synapse with the intrinsic properties of the postsynaptic neuron. We suggest that the richness of the dynamical properties of most biological neurons complicates the simple interpretation of the results of functional connectivity analyses using Granger causality.

## Background

The goal of much of neuroscience is to understand how circuit dynamics arise from the properties of the circuit neurons and their connectivity [1]. Unfortunately, the number of circuits whose anatomical connectivity is well-established is relatively low. Additionally, even when available, the connectivity diagram only provides a static description of synaptic connectivity, while in reality synaptic strength varies as a function of neuromodulation [2-5] and time-dependent processes such as facilitation and depression [1,6-8].

In most circuits, the anatomical connectivity is not completely known, and functional connectivity techniques are often used to give a directed, dynamically changing assessment of network interaction [9-14]. Functional connectivity is defined as the strength of the influence one network member has on another during ongoing circuit behavior [15,16]. Such a metric is desirable because it provides a tool to describe circuits in terms of their functional interactions rather than only the presence or absence of synaptic connectivity. Additionally, functional connectivity metrics are desirable because unlike static, anatomical descriptions, they can be used to measure changes in how network elements influence one another when repeated over time and over different biological states. In the present work, we apply functional connectivity analyses to a biological circuit, the pyloric network of the crustacean stomatogastric ganglion (STG) in which the anatomical connectivity is known [17] to study the relationship between these two network descriptors.

One method of functional connectivity analysis that is widely used in neuroscience is Granger causality (GC), a technique from the field of economics [18,19]. GC is based on the idea that, given two signals X and Y, if knowing the past of Y is useful for predicting the future of X then Y must have a causal influence on X. Because GC is capable of estimating directed interactions between multiple time series, it can be a useful tool for determining the functional strength and direction of connections between members of a neural network [10,19,20]. Granger causality has been applied to neural networks of diverse sizes and used to measure dynamically changing connectivity in functional magnetic resonance imaging (fMRI) learning paradigms [21], the interaction of closely related nuclei in a simulated hippocampus during environmental exploration [22], and the plastic changes in the connectivity of single neurons in a cortical cell culture [23,24]. With few exceptions [25-27], however, functional network estimations made with GC have not been directly compared with underlying anatomical connectivity because the connection pattern is either not known or highly complex.

In the present work we apply GC analysis to electrophysiological recordings and model data from the STG to study the relationship between functional and anatomical connectivity more rigorously. The STG has been a model system for studies of neuromodulation and motor pattern generation for many years [28-31]. One major advantage of the pyloric circuit is its small size, which allowed investigators to map its connectivity [17,32]. While the anatomical connectivity of the circuit is known, less is known about how each synaptic connection contributes to the functional output of the system, under many different modulatory conditions. This is complicated by the fact that every synapse in the pyloric circuit can be modulated by one or more amines and neuropeptides [3,28,33,34] and that they show synaptic depression [35]. Classically, synaptic strength between two neurons can be well-studied using electrophysiological methods when the network's activity is silenced [35,36], but it is more difficult to measure synaptic strength during ongoing network activity. Consequently, the effects of a neuromodulator can be effectively studied on single synapses [34,37], but it can be difficult to assess the functional importance of those changes when the network is active, and each neuron is influenced by many others in the circuit. For these reasons, we were interested to ask whether GC would be a useful tool with which to study functional connectivity during ongoing network activity.

## Results

**Figure 1:**
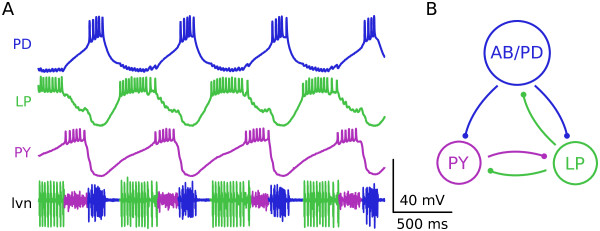
**Canonical pyloric triphasic rhythm**. **(A) **Simultaneous intracellular recordings from the core pyloric circuit neurons (top three traces) and an extracellular recording of a motor nerve (lvn) during an ongoing pyloric circuit oscillation. Cell abbreviations are: AB/PD (anterior burster/pyloric dilator, top trace, blue), LP (lateral pyloric neuron, middle trace, green), PY (pyloric neuron, bottom trace, purple). Spiking in all three cells can be measured concurrently on the lateral ventricular nerve (lvn, bottom row, colors demarcate spikes from each individual neuron and illustrate the temporal segregation of each neuron's spike times). **(B) **Simplified connectivity diagram of the pyloric circuit showing the individual neurons color coded as in (A) and the major synaptic connections between the cells. Notably, there is no synapse from the PY to the PD neuron. Synapses are also color coded as in (A) with the presynaptic cell determining color. All chemical synapses are inhibitory.

The crab STG has 26 neurons, about a third of which are considered part of the core pyloric circuit. The triphasic pyloric rhythm consists of rhythmic alternating patterns of activation in the two Pyloric Dilator (PD), single Lateral Pyloric (LP) and five Pyloric (PY) neurons, as seen in the recordings shown in Figure 1. Intracellular recording techniques coupled with cell deletions established the connectivity among these neurons in lobsters [17,32,38], and the core circuit elements are conserved in crabs (Figure 1). The pyloric rhythm depends on a pacemaker kernel consisting of a single anterior burster (AB) neuron coupled electrically to two PD neurons such that these three cells always fire together (shown schematically in Figure 1 as a single AB/PD neuron). The rhythmicity of the circuit comes from the bursting pacemaker properties of the AB/PD neuron pacemaker kernel [39], while the LP and PY neurons fire on rebound from inhibition with their timing determined by the strength of their inhibitory inputs and their intrinsic membrane currents [28,40-43]. All chemical synapses in the circuit are inhibitory.

### Functional connectivity computed with Granger causality differs from known anatomical connectivity

**Figure 2:**
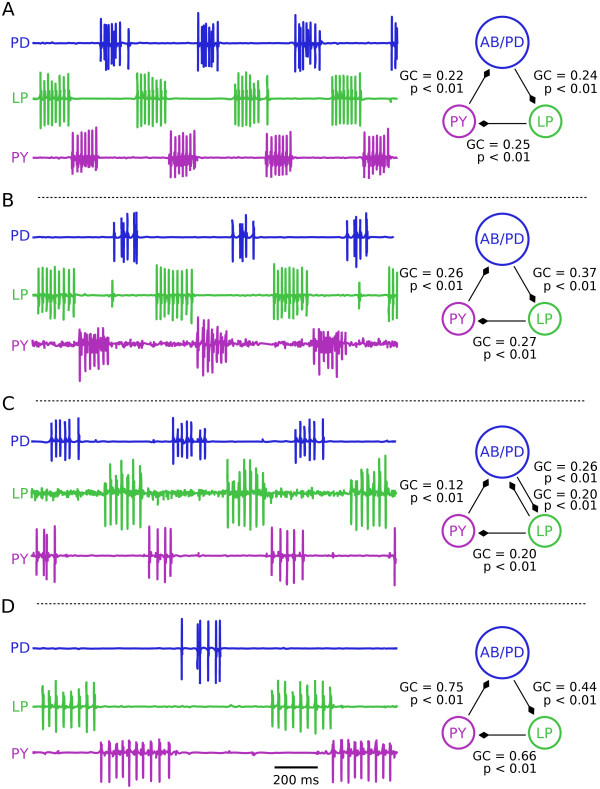
**Granger Causality analysis of biological stomatogastric ganglion (STG) circuits**. **(A-D)**. Extracellular recordings show the activity of the PD neurons (blue), the LP (green), and PY (purple) neurons from four different animals. Variability in period is visible between animals; but the phase relationships (relative times of firing) are maintained. Estimations of the functional relationships between the neurons using Granger causality (GC) are shown in the right panels and revealed a circular pattern of causal relationships in the network. Neurons are represented as circles and functional connections, as predicted by GC analysis, as lines between them with the diamond tips indicating the directionality of the connection. Each functional connection shown fell below the threshold for statistical significance of 0.05 (adjusted for multiple comparisons to 0.008). All parameters were kept constant during analysis across different data sets except for the total length of the input trace, ranging in these examples between 3 - 10 seconds of data. The auto-regressive model time lag parameter was fixed at 400 ms for all networks

Previous work has shown considerable animal-to-animal variation in the synaptic strength from the pacemaker neurons to the LP neuron [36]. Consequently, we were curious to determine if application of GC to pyloric rhythm recordings from different animals would show the same kind of variation across preparations. We applied GC methods to pyloric rhythms recorded from four different animals (Figure 2). All of these preparations show the canonical progression of PD, LP, and PY, and had periods and phase relationships typical of crab pyloric rhythms [36].

Granger values numerically represent how much the prediction of future values of a particular trace is improved by the inclusion of the past values of an additional trace. Mathematically, GC values are computed by taking the logarithm of the ratio of the prediction error when not considering the additional trace to the prediction error when considering the additional trace. This ratio is always greater than one (error can only decrease when considering more information) and thus the logarithm of this ratio, Granger causality, is always greater than zero. A Granger value of zero would represent a prediction error ratio of 1 (for example, no error reduction with the inclusion of more information, e^0 ^= 1) and a Granger value of 1 would represent a prediction error ratio of about 3 (for example, a 3 fold reduction in error, e^1 ^= 2.71). Typical Granger values from biological data sets were around 0.2 (e^0.2 ^= 1.2, prediction error reduced by about 20%) and for highly correlated traces (see below) around 1.0.

We prepared data sets as described in the Methods by computing a smooth rate function from spike-detected extra-cellular recordings and then applying the GC algorithm to each of these sets of recordings (Figure 2, color code as in Figure 1; in this paper, solid lines with diamond tips represent statistically significant Granger interactions). Because there was heterogeneity in the traces in terms of period length, number of spikes per burst and burst duration (Figure 2), we allowed the total data length analyzed to vary, but ensured that in all cases the total data length was at least 3 times the period. Further, we mandated that results were qualitatively insensitive to small changes in the data length. In this context, we defined parameter insensitivity to mean equivalent statistically significant Granger relationships when data length was changed by one period. Excepting total data length, all other parameters were kept constant between different data sets. Important among the constant parameters was the number of past time points that the GC algorithm would consider in constructing each auto-regressive model (the model order). If this time window was allowed to be too long, the auto-regressive model derived from a single trace would become highly effective at predicting its own future. This is because after an entire period has elapsed in an oscillator, a highly accurate auto-regressive model can be constructed. In this situation, additional external data sources can no longer aid in producing a more accurate model and thus are not considered to be Granger causal. For all biological data, we fixed the time lag parameter at approximately 40% of a pyloric period, or about 400 ms in keeping with the general principle of selecting model orders that adequately capture the timescale of the interactions of interest [19]. This time window captures the slow oscillation and resulting graded neurotransmitter release seen between cells during a pyloric cycle [44,45]. Importantly, graded neurotransmitter release (and thus causal interaction) due to the slow voltage oscillation is known to be functionally relevant in the pyloric circuit [45,46].

Statistically significant functional connections were found with GC from the PD to the LP neurons and from the LP to the PY neurons, both connections that are known to have an anatomical analog in biological preparations (Figure 1). Notably, GC operated successfully on these data even though all neurons are coupled by inhibition. In all four data sets (Figure 2A-D), there is a Granger causal relationship from the PY to the PD neuron. While it is true that PY neuron activity always precedes PD neuron activity in the temporal progression of the pyloric rhythm, the PY neurons do not directly inhibit the PD neurons and thus a functional connection cannot be related to direct neurotransmitter release from PY to PD. Importantly, we had selected a model order of 400 ms, a window of time too short to allow for GC to report functional interactions from PY to PD related to the disynaptic connection via the LP neuron. The consistency of the functional connection between the PY and PD neuron between datasets led us to hypothesize that in data sets with strong oscillations, where temporally structured relationships between neurons may not necessarily be due to direct synaptic interactions, a fundamental confound may emerge in interpreting functional connectivity computed with Granger causality.

Given the functional connection between two neurons that was not attributable to a known synaptic connection, we set out to account for this unexpected finding by analyzing a variety of artificial data (with known properties), to gain intuition for what classes of data sets lend themselves well to GC and under what conditions the analysis could break down.

### Granger causality matches known functional relationships in synthetic anti-correlated data

**Figure 3:**
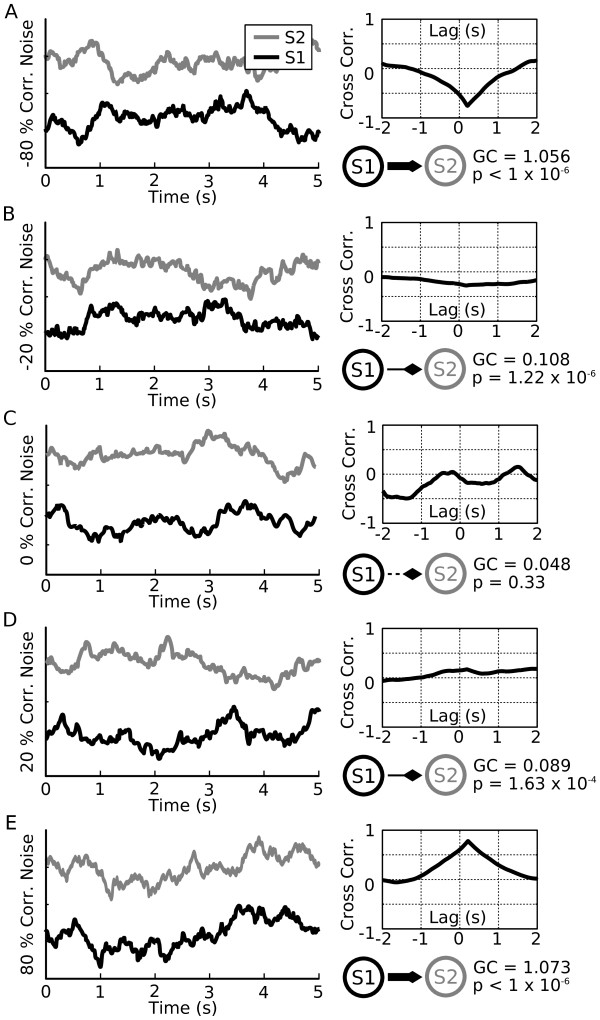
**Granger Causality analysis correctly identifies the relationship between correlated noise. (A - E).** Left panels show randomly generated correlated noise (A - E, black and gray lines, S1 and S2 respectively). Correlation values ranged between perfectly anti-correlated (-100%) to perfectly correlated (+100%). In all cases (A - E, panels plot traces with correlation values of -80%, -20%, 0%, 20% and 80%), an artificial delay of 200 ms is introduced into the correlation from the black trace (S1) to the gray trace (S2). The cross-correlation (A - E, top right panels) shows a peak proportional to the amount of correlation used to generate the noise and a peak offset by the delay value of 200 ms. For each value of correlation, we compute the Granger causality (A - E, bottom right panels). Schematic network diagrams summarize the predicted Granger causality (GC) values by the thickness of the lines from S1 to S2 and diamond tips indicate the direction of the causal relationship. In all cases where a significant GC value is computed, S2 is Granger caused by S1 as expected by the time lag relationship. Note that for 0% correlation (for example, two independent noise traces), the computed GC value does not fall above the threshold for significance (C, bottom right panel, *P *= 0.33, dashed line). For either positive or negative correlation at 20% the computed GC is significant (B: -20%, *P *= 1.22 × 10^-6^; D: +20%, *P *= 1.63 × 10^-4^). For 80% correlated noise GC is highly significant (A: -80%, *P *< 1 × 10^-6^; E: +80%, *P *< 1 × 10^-6^).

We generated synthetic data sets with predetermined causal relationships. We first generated paired traces of correlated noise using two Ornstein-Uhlenbeck processes [47] that were related by a predetermined correlation value -1.0 ≤ *c *≤ 1.0. Using this approach we generated random time series that ranged from perfectly anti-correlated (*c *= -1.0) to statistically independent (*c *= 0.0) to perfectly correlated (*c *= 1.0). We specifically included anti-correlated noise in the analysis because neurons in the STG are coupled with inhibitory synapses and thus we wanted to determine whether anti-correlation (which can be viewed as conceptually similar to synaptic inhibition) would be correctly recognized as a causal interaction. Additionally, we generated the time series in such a way that any correlation we introduced was offset by a time lag of 200 ms. In this manner we were able to introduce a 'causal' relationship between the two traces as the similarity (or anti-similarity) between the signals would be offset by the predetermined time lag of 200 ms. When using high levels of correlation, the noise traces looked qualitatively similar (Figure 3A and 3E, negative and positive correlation of 80% respectively). For lower correlation values the relationship between the signals was less obvious to visual inspection (Figure 3B and 3D, negative and positive correlation of 20% respectively) but emerged when the cross-correlation between the two traces was computed (Figure 3A-E, top right panels). For non-zero values of *c*, the cross-correlation curve showed a peak proportional in height to *c *and in all cases was offset by a time lag of 200 ms.

The correlated noise traces were then used as inputs to the GC algorithm to test how GC would change given different levels of correlation and anti-correlation between the two traces. In the case of zero correlation (that is, independent noise traces) the computed value of GC did not meet the requirements for significance and thus we concluded that there was no GC relationship as expected (Figure 3C, network diagram, GC = 0.048, *P *= 0.33, in this paper, lines are drawn as dashed to indicate the lack of statistical significance). When the correlation value was changed to either -20% or +20% significant GC values were obtained (Figure 3B, *c *= -0.2, GC = 0.108, *P *= 1.22 × 10^-6^; Figure 3D, *c *= 0.2, GC = 0.089, *P *= 1.63 × 10^-4^). Finally, when correlation values were raised to *c *= -80% and *c *= +80% highly significant GC values were obtained (Figure 3A, *c *= -0.8, GC = 1.056, *P *< 1 × 10^-6^; Figure 3E, *c *= 0.8, GC = 1.073, *P *< 1 × 10^-6^). Diamond-tipped lines are drawn with a thickness proportional to the strength of the GC relationship between S1 and S2. Thus, GC analysis detected directed relationships in correlated noise and the computed GC values were larger for traces that were more strongly correlated. Furthermore, GC analysis was equally effective at detecting functional relationships between negatively correlated traces (an 'inhibitory interaction') and positively correlated traces (an 'excitatory interaction').

When analyzing data of this nature an advantage of GC over cross-correlation analysis is that GC is less sensitive to variability especially at lower levels of correlation. While the peaks of the cross-correlation functions matched the predetermined values of correlation in the examples shown, in general the cross-correlation curves tended to have a large amount of variability. This is evident in the cross correlation curve of the two fully independent noise traces (Figure 3C, top right plot) which reports correlation values as high as -50% attributable to chance. If cross-correlation functions from repeated trials were averaged, the peak in the averaged function would converge upon the input value (data not shown). In contrast, GC analysis reports a highly significant relationship between the two traces correlated at only ± 20% (Figure 3B and 3D, network diagram) from a single trial. We hypothesize that this is due to the fact that GC considers a window of data over the entire data trace (thus implicitly averaging) while cross-correlation is a point-wise measure and thus sensitive to small fluctuations.

A disadvantage of GC is that it does not report the time lag between the two input waveforms, information that is reconstructed by sequentially taking the cross-correlation over the entire range of time lags. Time lag information could be extracted from GC analysis by looking at the coefficients of the auto-regressive model. In these examples, the largest coefficients (the most predictive past values of each trace) would fall at the artificially introduced time lag of 200 ms.

### Granger causality matches synaptic architecture in model neuron inhibitory circuits

**Figure 4:**
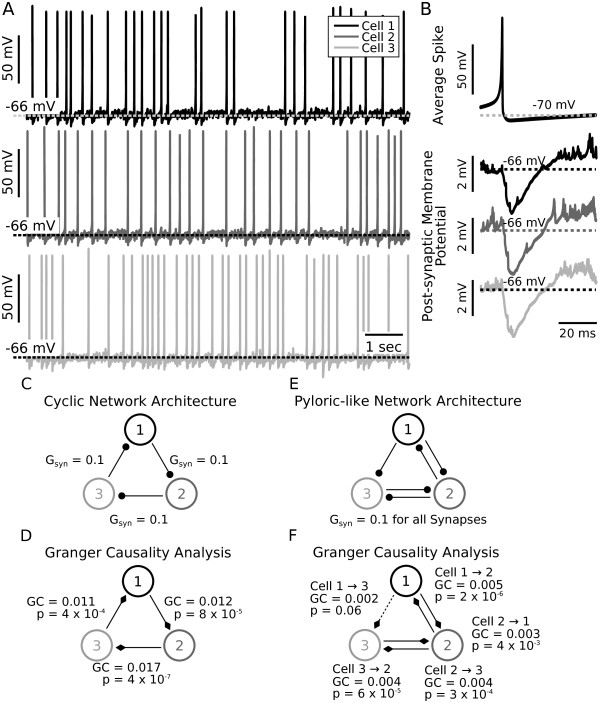
**Granger Causality analysis correctly identifies the inhibitory relationship between model neurons**. **(A) **Three quadratic integrate-and-fire Izhikevitch neurons [48] were coupled in a cyclically inhibitory fashion (network topology schematic in panel C) and made to fire slowly and with variability (rate = 2.5 Hz with CV = 0.6, black, dark gray, light gray traces correspond to individual model cells). **(B) **Traces show an average spike (top panel) and average voltage waveforms in each cell in a 60 ms time window triggered on a spike occurring in the presynaptic neuron (bottom three panels). Trace gray scales correspond to the legend in A. **(C) **Network layout includes three cells that inhibit each other in a cyclical fashion. Synaptic maximal conductances are all uniformly set to 0.1 nS. Filled circles indicate inhibition. Gray scales of cells (numbered 1, 2, 3) correspond to the legend in A. **(D) **Granger causality analysis predicts a causal relationship closely matching synaptic architecture. Filled triangles indicate the directionality of the statistically significant Granger causal relationships between cells. Granger values and *P*-values are indicated in the figure. **(E) **Pyloric-like network topology comparable to that shown in Figure 1B. Synaptic coupling between cells 1 and 2 is reciprocal as is the synaptic coupling between cell 2 and cell 3. Cell 1 additionally inhibits cell 3 but cell 3 does not synapse onto cell 1. **(F) **Granger causality analysis succeeded in reconstructing this network architecture. Statistically significant Granger values were found for all synaptic connections between cells 1 and 2 as well as for cells 2 and 3. For the synapse from cell 1 to cell 3 the Granger value came close to the threshold for statistical significance (line drawn as dashed to indicate lack of significance). The Granger value for the non-existent synapse from cell 3 to cell 1 (not drawn) was non-significant (*P *= 0.33).

We next wondered if Granger causal functional connections between model neurons connected with inhibition would match known underlying synaptic architecture. To test this we constructed a three neuron inhibitory network consisting of quadratic integrate-and-fire Izhikevitch neurons [48]. We chose Izhikevitch model neurons because they are a generic representation of neuronal features and because simulation results using such model neurons are unlikely to be due to model-specific properties. Each neuron was individually made to fire with moderate variability by injecting noisy input current (Figure 4A, sample spike trains, mean rate 2.5 Hz, CV = 0.6). This variable firing pattern was chosen to distinguish the resulting voltage traces from pyloric recordings in which cells fire in a much more structured, oscillatory pattern. Model cells were first coupled in a circular fashion. Thus, cell 1 inhibited cell 2, cell 2 inhibited cell 3 and cell 3 inhibited cell 1 (Figure 4B hyperpolarizing voltage deflections due to inhibitory synaptic input; Figure 4C cyclic network diagram, all model synapses had equal maximal conductances of 0.1 nS).

As expected, the effect of this coupling pattern was that spikes in each cell evoked hyperpolarizations of a few mVs in the respective postsynaptic neuron, as shown in spike-triggered averages (Figure 4B, traces have same grayscale as in A). This indicates that, as expected, spikes hyperpolarized the membrane voltage of target cells and thus could be considered to be causally influencing the spike times of their post-synaptic partner cells.

Spike trains were first turned into binary spike trains and then convolved with a half-Gaussian (see Methods) to produce a smooth rate function (as was done previously with the experimental data). In these data, functional connectivity computed with GC matched the synaptic structure of the network (Figure 4C). Although each cell was firing slowly and with variability, the GC relationship reported between each cell pair was highly significant (Figure 4D, Cell 1 to 2: GC = 0.012, *P *= 8.11 × 10^-5^; Cell 2 to 3: GC = 0.017, *P *= 3.98 × 10^-7^; Cell 3 to 1: GC = 0.011, *P *= 3.82 × 10^-4^).

Notably, because the input data are based upon an approximation of a rate function, they contain none of the subthreshold voltage deflections directly caused by inhibition. Thus, causal interactions are being calculated purely based on the timing of spikes. This is significant because it suggests that the analysis is insensitive to whether inhibition or excitation mediates interactions between neurons as long as an appropriate model order is chosen. Indeed, when the same cyclic network was simulated with excitatory synapses, GC analysis produced equivalent output (data not shown). When using excitatory coupling instead of inhibition, the same connections were found to have statistically significant Granger values (values were: Cell 1→2: 0.012, Cell 2→3: 0.017, Cell 3→1: 0.011, *P *< 0.01 for all three numbers).

We also wondered what functional relationships would exist if the model network was not connected in a cyclical fashion but instead connected analogously to the pyloric circuit (Figure 1). This result was of interest because we wanted to know whether the functional interactions we observed in biological data (Figure 2), that matched anatomical synaptic connections (PD/LP and LP/PY), were actually based on the underlying synapses or whether those functional interactions were also a result of fixed temporal relationships due to oscillations in the spike trains (as we hypothesized for PY to PD). To answer this, we reasoned that in a network coupled analogously to the pyloric circuit, but in which cells fired with variability (that is, without oscillations), there should be no functional interaction between the PY and the PD cell while the PD/LP and the LP/PY interactions ought to remain. Thus, we coupled the same model cells (Cells 1, 2 and 3) as in the pyloric circuit in which the PD/LP pair and the LP/PY pair are connected with reciprocal inhibition while PD inhibits PY without a return synapse from PY to PD. For all synapses we used synaptic maximal conductances of 0.1 nS (Figure 4E). Statistically significant GC relationships were found between the cell 1/2 pair (analogous to PD/LP) and the cell 2/3 pair (analogous to LP/PY) all of which match the synaptic architecture (Figure 4F, GC and *P *values given in the figure). Additionally, a functional connection from cell 1 to cell 3 was present but fell slightly above the threshold for significance (Figure 4F, cell 1 to 3, GC = 0.002, *P *= 0.06, dashed line indicates lack of significance). Importantly, the GC value for the PY to PD synapse was far from achieving statistical significance (GC = 0.001, *P *= 0.33). Thus, we conclude that due to the lack of oscillatory activity in this model, functional connections matched synaptic architecture even when model cells were coupled analogously to the pyloric circuit. This finding reinforces the hypothesis that functional connections unrelated to synaptic connections seen in the biological data (Figure 2) are related to the strongly oscillatory behavior of the AB/PD neurons.

### Granger causality does not correlate with synaptic strength in a set of pyloric network models containing an oscillator

To investigate the relationship between synaptic conductance magnitude and the strength of the functional relationships we applied GC analysis to a set of 84 pyloric model networks with highly variable intrinsic and synaptic properties. These 84 networks are a subset of a much larger pool of model networks [49] that had a wide range of known parameters in their synaptic conductances and membrane conductances. To further increase the variability of synaptic and intrinsic conductance values in the selected model networks we introduced a kinetic temperature dependency into each synapse and ion channel and then simulated each network at different temperatures (see Methods). Therefore, a large variety of synaptic conductances and channel time constants was represented by the selected model networks.

**Figure 5:**
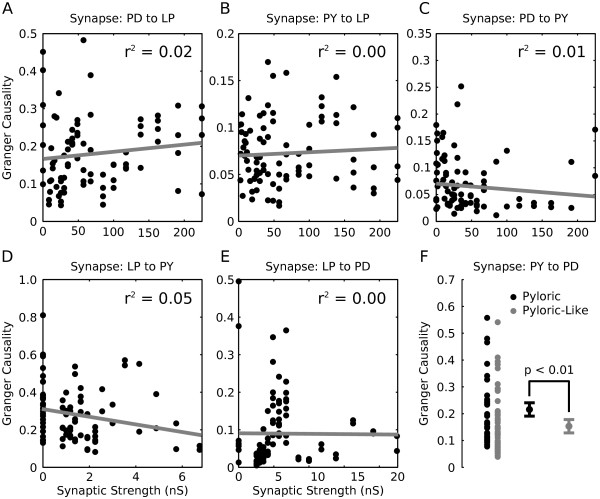
**Granger causality values do not correlate with synaptic strengths in a set of pyloric oscillator model networks**. We selected a set of 84 model networks all of which displayed representative pyloric-like outputs and which had diverse underlying biophysical implementations in terms of their ionic and synaptic conductances. For each synapse between the three model cells (PD, pyloric dilator; LP, lateral pyloric; PY, pyloric neuron) we computed a Granger causality (GC) value (panels A - E). We then plotted this value against the synaptic strength given to that connection in that network (black dots each represent the values from a single network). We found no significant linear relationship between synaptic strength and GC value for any of the five synapses (r^2 ^values ranged from 0.00 - 0.05). Panel F shows the GC values predicted for the PY to PD synapse which is known to have no anatomical analog (maximal conductance for that synapse was 0.0 in all model networks). We separated the networks into two groups: pyloric (black dots) and pyloric-like (gray dots) and found that the pyloric group had a significantly higher GC values on average (Kruskal-Wallis test, *P *= 0.0044, n_pyloric _= 29, n_pyloric-like _= 55, mean_pyloric _= 0.22, mean_pyloric-like _= 0.15, error bars are standard error of the mean (SEM)).

From each model network we took voltage traces, computed a smooth rate function and the GC value corresponding to each possible pair-wise interaction between the three neurons in the pyloric microcircuit (PD, LP, PY). We then plotted those numbers against the maximal synaptic conductances for each model synapse for each individual network (Figure 5). Surprisingly, we found a large range of computed GC values for each value of synaptic conductance. While previous studies suggested a complex, non-linear relationship between synaptic strength and GC value [24] we observed no relationship between GC value and synaptic strength for any of the synapses (Figure 5). We hypothesize that, because GC collapses the effect of synaptic conductance with the modulation of the postsynaptic neuron into a single number (representing functional strength), we do not see a relationship between synaptic conductance and functional connectivity. Granger causality captures the total strength of an interaction but cannot disambiguate between synaptic and postsynaptic contributions. Because of this, care must be taken when using GC to make inferences about underlying synaptic architecture.

As part of the GC calculations, we computed GC values for the PY to PD synapses in all 84 networks. It is known that this synaptic connection does not exist in biological preparations (Figure 1) and thus, by design, g_syn _for the model PY to PD synapse was zero in all model networks considered in this analysis. Nonetheless, many networks had GC values for the PY to PD interaction that were comparable to values computed for the other synapses (Figure 5F, PY to PD).

Previously, when considering biological data (Figure 1) we attributed functional connections unrelated to synaptic connectivity to the highly oscillatory nature of pyloric activity. We hypothesized that a functional relationship existed due to the fixed temporal relationship between the PY and PD neurons. To test whether this was the case in the set of model pyloric oscillators we subdivided the 84 networks into two groups, those that displayed fully pyloric behavior (triphasic, correct burst order, with appropriate phase relationships and burst durations [49]) and those that were pyloric-like (triphasic but with abnormally long burst durations, tonic firing instead of bursting, or phase relationships not seen in normal biological data). The mean GC values of the two populations were significantly different from one another (Figure 5, bottom right panel, *P *< 0.01, Kruskal-Wallis test, n_pyloric _= 29, n_pyloric-like _= 55). GC values for the PY to PD synapse were significantly higher when regular oscillations are present (Figure 5, black dots, pyloric networks only) as compared with more diverse firing behavior (Figure 5, gray dots, pyloric-like networks only) providing support for the conclusion that oscillations can lead to functional connectivity unrelated to synaptic connectivity.

## Discussion

While it is well appreciated that functional connectivity methods are not equivalent to direct anatomical and physiological measures of connectivity, it is clear that the two are related [19]. The nature of the relationship is difficult to determine because the strength of a biological synaptic connection will be altered by the synapse's history of activation and neuromodulation, and the ability of a synaptic input to influence the firing of its target neuron will be altered by changes in the intrinsic postsynaptic excitability. Reliable functional connectivity metrics would be extremely useful because they can be used during ongoing circuit performance to assess how important each synapse is to the circuit's output under different conditions. Here we demonstrate that Granger causality, a measure of functional connectivity, can be used with synaptic inhibition, unlike previous claims to the contrary [24]. Further, we point out a serious limitation in interpreting results of Granger methods when there are strongly oscillatory components in the data. The ability of an input to influence a target neuron's activity depends both on the input strength and the target neuron's excitability. These two factors are effectively collapsed in Granger calculations leading to ambiguity about the mechanism underlying a changed functional connection.

We used the pyloric network of the stomatogastric ganglion as the test circuit for these studies, precisely because the mechanisms underlying the generation of the pyloric rhythm are well understood [28]. Rhythm generation in the pyloric circuit depends on a strongly oscillatory pacemaker kernel of three electrically coupled neurons (the AB and two PD neurons) [39], and the specific pattern of firing depends on the postinhibitory rebound properties of the LP and PY neurons [41,42], as all of the synaptic connections among the pyloric circuit neurons are inhibitory (Figure 1).

Based on GC analysis, a functional relationship exists from the PY neuron to the PD neuron in biological extracellular recordings, an interaction which is not based on a synaptic connection in biological networks. We argue that this functional connection arises from the highly regular oscillations displayed by the circuit that causes the PY and PD neurons to have a fixed, repeating temporal relationship that is not related to their direct interaction. In other words, in each data set, the PD neurons, because of their intrinsic oscillation period, always fired with a predictable latency after the PY neurons, although the onset of PD activity was in no way causally related to the PY neuron activity. We provided support for this hypothesis by generating a model network that had pyloric-like connectivity but in which the neurons fired with variability instead of in a regularly oscillating pattern (Figure 4E, F). In this case, functional relationships matched the synaptic architecture closely. The interpretation of our results that oscillations introduce functional relationships unrelated to synaptic architecture is further strengthened by the higher Granger values computed for the PY to PD synapse in regularly oscillating pyloric networks when compared to networks that have pyloric-like activity, but are not reliably oscillatory (Figure 5).

Given the prevalence of oscillations in the brain [50-53] and the confound posed by these oscillations for interpreting GC analyses, care should be taken when applying functional connectivity metrics to neuronal data. Neuronal oscillations could complicate the interpretation of data gathered with electroencephalography (EEG) [11] or other methods [22,54]. Presumably, the stronger and more regular the oscillation, the more serious this potential confound is likely to be. Certainly, in many brain areas oscillations are less regular and weaker than those found in the pyloric rhythm. Nonetheless, given the widespread use and appeal of GC methods, it is important to recognize that oscillations in the data could lead to functional connectivity unrelated to synaptic interactions.

Granger causality is one method in a family of functional network estimation methods each with its own advantages and drawbacks. GC, which is based on auto-regressive modeling [18,19], can also be computed in the spectral domain where one GC value is computed for each frequency [55,56]. This can be useful for separating the interactions of signals that contain multiple frequency components. Additionally, the concept of partial Granger causality provides methods for removing the effects of external inputs common to all network members or unmeasured network elements [57]. Throughout this study, we compared our calculations with those returned by partial Granger causality and found no difference in the resulting values. We selected standard GC for this study because of the maturity of the existing tools [19] and the large set of previous studies that have employed the method. Other related methods include dynamic causal modeling that relies on Baysian inference to measure the causal interactions between signals [58] or Transfer Entropy that relies on mutual information to compute interactions between network members [59]. We suspect that many measures of functional or effective connectivity will share the same potential interpretation issues as those outlined here. These issues in no way preclude the utility of these methods for understanding how different brain regions interact, as long as mechanistic conclusions are not made from them.

Inhibition is critically important in all brain regions and all functional brain circuits. We show here that GC works equivalently with inhibition and excitation. Importantly, if one uses test model neurons that are relatively silent unless driven by excitation and have no postinhibitory rebound, then a functional relationship may not appear because the postsynaptic neurons are not firing. However, if the postsynaptic neuron is either tonically active unless inhibited, or has postinhibitory rebound [60], then the change in the postsynaptic neuron's firing will form the basis for a functional interaction. Given that postinhibitory rebounds can be either relatively short or quite long [61], care should be taken to ensure that an appropriate model order is used to capture potential rebound firing.

In previous reports GC was found to be well-correlated with the amplitude of the synaptic potential [24]. In contrast, we found little correlation between GC and synaptic strength in a population of neuronal models (Figure 5). This result may, at first, seem puzzling. Nonetheless, it is important to remember that the spike timing of a postsynaptic neuron will always be an interaction between the strength of the input synapse and the intrinsic properties of the follower neuron [1]. The population of models studied in Figure 5 contains model pyloric networks with highly variable synaptic and intrinsic conductances [49]. Therefore, it is likely that the variation of synaptic strength is compensated by variations in the intrinsic membrane properties of the follower neurons [36,49,62], as synaptic and intrinsic parameters can compensate for each other in a functional network [63]. We conclude that because Granger causality represents the total effect of one cell on another, it effectively collapses the relative contribution of synaptic strength with any postsynaptic modulation due to intrinsic properties. Thus, relating Granger causality directly to synaptic strength is not possible as has been previously noted [19], even if the two numbers are related.

While we did not observe a relationship between pyloric synaptic strength and functional coupling strength (Figure 5) we did, however, observe such a relationship in the correlated noise data (Figure 3) and spiking model data (data not shown). This is consistent with the interpretation that the complex membrane properties of the follower neurons in the pyloric networks account for the lack of correspondence between synaptic conductance and GC values. Because many real biological neurons in the brain express complex membrane properties [64-68] that are not well-captured by simple integrate and fire or rate models (such as those used in many tests of the functional connectivity algorithms), this will potentially complicate the simple interpretation of GC for biological data. In particular, changes in neuromodulatory tone across the brain may alter both target neuron excitability and the strength of the inputs to the target. Thus, Granger causality may show that two brain regions are more 'effectively connected' when the strength of the input itself may be unchanged or even decreased.

We also found that functional interactions computed from spike timing information alone could mirror synaptic architecture. In our spiking model data the inhibitory interactions of the neurons caused hyperpolarizing deflections in post-synaptic membrane voltages (Figure 4) but this information was filtered out when spike trains were smoothed before GC analysis leaving only the relative timing of spikes. Functional relationships based on spike timing information matched synaptic architecture even when model synaptic connectivity was more complicated as in the pyloric-like model network (Figure 4E). In this network, some synaptic interactions are reciprocal and single cells receive multiple inputs that can lead to complicated interactions. Computing functional interactions that match network topology from spike timing information alone is non-trivial and speaks to the utility of the Granger method. Functional connection patterns based on spike times that closely match network topologies could also be useful for *in vivo *data from behaving animals that, similar to our recordings in the pyloric circuit, rely heavily on extracellular recordings of spike times. Spike times were recently shown to be sufficient to fully mathematically infer synaptic weights in a small network of integrate and fire neurons [26,69].

## Conclusions

We investigated the utility of Granger causality analysis for understanding functional interactions in a rhythmic, inhibitory circuit with a known connectivity diagram. We found that oscillations in the input data can yield functional connections that are not predicated on biological interactions. Additionally, we found that GC functions well in the context of inhibitory coupling and that when postsynaptic neurons have complex and realistic intrinsic membrane properties, the relationship between GC results and actual synaptic strength becomes more complicated.

## Methods

### Synthetic data

Noise waveforms were generated as Ornstein-Uhlenbeck processes [47] with an iterative update rule described in detail in Destexhe et al. [70]. Briefly, each trace is a random walk, *g*, given by:

where g_0 _is the mean value of the process, Δ*t *is the time step, *τ *is the time constant of the random walk, *r*_*norm *_is a normally distributed random number with 0 mean and unit standard deviation and the amplitude coefficient, *A*, is given by:

where *D *is the diffusion coefficient. During each time step the random number for the first trace, *r*_1_, is generated *de novo*. This number is saved until 200 ms of simulation time have passed and then used to compute a second random number, *r*_2_, for use in the second noise trace subject to the following modification:

where *c *is the desired level of correlation between the two traces and *r*_*norm *_is a number from the normal distribution with 0 mean and unit standard deviation. Cells in the three cell noisy firing model network are Izhikevitch neurons [48] with voltage waveforms governed by:

with the reset condition:

where parameter values are *a *= 0.02, *b *= -0.1, *c *= -55, *d *= 6, *I*_*app *_= 4.65 where *I*_*app *_represents the applied current. Synapses are modeled as double exponential waveforms [71] with *τ*_*rise *_= 0.7 and *τ*_*fall *_= 5.8.

### STG model simulations

Simulation of STG networks followed established approaches [49]. All model neurons were selected from an existing database of STG cells [72]. Twelve total networks were selected such that the output voltage trace displayed either fully pyloric behavior or pyloric-like behavior. Fully pyloric behavior was defined by phase, duty cycle and relative burst times of the three cells that matched *in vitro *recordings [49]. Pyloric-like networks had burst frequencies, spike rates or phase relationships that fell outside of the normal range of behaviors observed in biological preparations. Synaptic strengths between model cells could take on values of 0, 3, 10, 30, 100, or 200 nS. Each network was run over a range of temperatures (7, 11, 15, 19, 23, 27, or 31°C) to yield a total of 84 different networks. Each ionic conductance and each synapse was subject to kinetic modification based on the temperature of the simulation [73]. Conductances were updated based on the equation  where Q_10 _= 1.5, R_2 _is the updated conductance, R_1 _was the existing value of the conductance, T_2 _was the simulation temperature and T_1 _was the base temperature set to 11°C. This equation was solved for R_2 _to yield . All activation and inactivation rates were given Q_10 _= 2. Logarithms are natural logarithms (base *e*). After simulation, voltage traces were preprocessed and analyzed with Granger causality in the same manner as all other data sets.

### Animals/Dissection

*Cancer borealis *were purchased from Commercial Lobster (Boston, MA, USA) and maintained in artificial seawater tanks at 10 - 12°C. Before dissection, animals were cold-anesthetized by packing them in ice for 30·min. The stomach was removed from the crab and placed in chilled physiological saline NaCl, 440 mM; KCl, 13 mM; MgCl_2_, 26 mM; CaCl_2_, 13 mM; Trizma base, 11 mM; maleic acid, 5 mM; pH·7.45) while the stomatogastric nervous system (STNS) was dissected and pinned to a Sylgard-coated petri dish containing saline [74]. The stomatogastric ganglion (STG) was desheathed to allow for intracellular recording of the cell bodies.

### Electrophysiology

Extracellular recordings were obtained by making petroleum jelly wells around branching nerves of the STNS and placing a stainless steel electrode inside the well and one in the bath. The extracellular traces were amplified and recorded with an AM Systems Model 1700 AC Amplifier. During recordings, preparations were continuously superfused with chilled physiological saline (12°C) by means of a Peltier cooling system (Warner Instruments/Harvard Apparatus Hamden, CT) and the temperature was monitored using a thermoelectric probe in the bath.

### Data acquisition

All traces were recorded by a PC running pClamp software (Version 10.2). Spike times were extracted from the files using Spike 2 software (version 6.04) and a thresholding script. Data files were converted to a MATLAB compatible format for further processing.

### Data analysis

**Figure 6:**
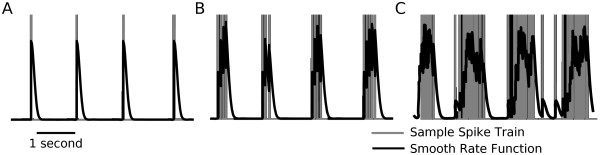
**Convolution of binary spike trains with a half-Gaussian is used to prepare data for analysis with Granger causality**. Illustration of the transform applied to spiking data before running Granger causality analysis. Several example spike trains are shown in gray (**A**: regular spiking, **B**: slow bursting, **C**: fast bursting). In general, raw voltage traces were rasterized (that is, converted to a series of delta functions at the time of each spike) and then convolved with a half-Gaussian function. This converts a point process into a smoother rate function (black traces) that is better suited to Granger causality analysis.

Final analysis was done in MATLAB (The Mathworks) with custom written scripts. Granger causality analysis was done with v2.8 of the toolbox described in Seth [19] which additionally provides a detailed description of the algorithm. Prior to Granger causality analysis, extracellular recordings and model data were all converted to continuous rate functions because the GC algorithm is designed for use with continuous waveforms [19]. More recently, methods to apply Granger causality directly to point processes such as spike trains have been proposed [75]. To achieve this we first converted voltage traces into binary spike trains which were then convolved with a half-Gaussian with only a rightward tail (Figure 6, black traces). The half-Gaussian was chosen because spikes cannot influence other cells before their time of occurrence. Using a half-Gaussian kernel ensured that a spike only contributed to the rate function after it had fired. If spikes occurred spaced out in time, the individual half-Gaussians are visible in the rate function (Figure 6A). If spikes were closer together in time, as in burst discharges, the half-Gaussians can temporally sum (Figure 6B,C). In general, we applied this process to all voltage recordings to produce optimal input data for the GC algorithm.

GC analysis, as any data analysis method, makes several assumptions about the data upon which it operates. We ensured that for all data sets analyzed, both biological and model, these assumptions were not violated. First, GC requires that input data are covariance stationary meaning that the mean and variance cannot change over time. The pyloric circuit is a canonical example of covariance stationarity in biological data. Second, the auto-regressive models that are constructed and form the basis of GC analysis must represent the data well. For all data we verified that the root mean square error in the models did not exceed 5% and in many cases model fits were significantly better.

Important parameter choices were the total length of the input data and the model order (the total number of past observations used to construct the auto-regressive model). Total input data length was varied between data sets but constrained to always include at least three oscillation periods. Excepting total data length analyzed, all other parameters were kept constant between different data sets. Model order was selected manually to best reflect the time scale of functional interactions in the data. For simulations of spiking neurons, for example, model order was chosen to span 100 ms, to capture the kinetics of the synaptic waveform. For pyloric data, a model order of 400 ms was used to capture a significant portion of the slow oscillation. Statistics were computed using the Kruskal-Wallis test. Plots were produced in MATLAB and then finished in the Inkscape (http://inkscape.org) graphics program.

## List of abbreviations

AB: anterior bursting neuron; CV: coefficient of variation; EEG: electroencephalography; fMRI: functional magnetic resonance imaging; GC: Granger causality; LP: lateral pyloric neuron; lvn: lateral ventricular nerve; PD: pyloric dilator; PY: pyloric neuron; SEM: standard error of the mean; STG: stomatogastric ganglion; STNS: stomatogastric nervous system;

## Competing interests

The authors declare that they have no competing interests.

## Authors' contributions

TK and GG planned this study. TK performed the Granger analysis and simulations, GG collected the electrophysiological data in Figure 2. TK and EM wrote the manuscript, and all authors contributed editorial revisions. All authors read and approved the final manuscript.
